# Positive *BRAF*V600E mutation of primary tumor influences radioiodine avidity but not prognosis of papillary thyroid cancer with lung metastases

**DOI:** 10.3389/fendo.2022.959089

**Published:** 2022-11-02

**Authors:** Shuhui Huang, Mengfang Qi, Tian Tian, Hongyuan Dai, Yuan Tang, Rui Huang

**Affiliations:** ^1^ Department of Nuclear Medicine, West China Hospital of Sichuan University, Chengdu, China; ^2^ Department of Pathology, West China Hospital of Sichuan University, Chengdu, China

**Keywords:** papillary thyroid carcinoma, BRAFV600E mutation, lung metastases, radioiodine avidity, prognosis

## Abstract

**Purpose:**

This study investigated the relationship between *BRAF*V600E mutation of the primary tumor and radioiodine avidity in lung metastases (LMs) and then further evaluated the impact of *BRAF*V600E mutation and radioiodine avidity status on the prognosis of papillary thyroid cancer (PTC) with LMs.

**Methods:**

Ninety-four PTC patients with LMs after total thyroidectomy and cervical lymph node dissection between January 2012 and September 2021 were retrospectively included. All patients received *BRAF*V600E mutation examination of primary tumors and radioactive iodine (RAI) therapy. The therapeutic response was evaluated by Response Evaluation Criteria in Solid Tumors (RECIST) assessments (version 1.1). For patients with target lesions, the response was divided into complete response (CR), partial response (PR), stable disease (SD), and progressive disease (PD); for patients without target lesions, the response was divided into CR, non-CR/non-PD, and PD. In therapeutic response, PR and SD were classified as non-CR/non-PD for analysis. The chi-square test and logistic regression were used to analyze the impact factor on PD and mortality. Progression-free survival (PFS) and overall survival (OS) curves were constructed by the Kaplan–Meier method.

**Results:**

It was found that 21.2% (7/33) of patients with positive *BRAF*V600E mutation and 62.3% (38/61) of patients with negative *BRAF*V600E mutation had radioiodine-avid LMs (χ^2^ = 14.484, p = 0.000). Patients with positive *BRAF*V600E mutation are more likely to lose radioiodine avidity; the odds ratios (ORs) were 5.323 (95% CI: 1.953–14.514, p = 0.001). Finally, 25 patients had PD, and six patients died; loss of radioiodine avidity was the independent predictor for PD, and the ORs were 10.207 (95% CI: 2.629–39.643, p = 0.001); *BRAF*V600E mutation status was not correlated with PD (p = 0.602), whether in the radioiodine avidity group (p = 1.000) or the non-radioiodine avidity group (p = 0.867). Similarly, *BRAF*V600E mutation status was not correlated with mortality; only loss of radioiodine avidity was the unfavorable factor associated with mortality in univariate analyses (p = 0.030).

**Conclusion:**

Patients with LMs of PTC were more likely to lose radioiodine avidity when their primary tumor had positive *BRAF*V600E mutation; however, only radioiodine avidity and not *BRAF*V600E mutation status affected the clinical outcome of patients with lung metastatic PTC.

## Introduction

Differentiated thyroid cancer (DTC) is the most common endocrine malignancy, accounting for 90% of all thyroid cancers (1). Although most cases of DTC can be curable with a favorable prognosis, 5% to 25% of patients still have distant metastases (DMs), with a 10-year survival rate of only approximately 50% (2, 3). Radioactive iodine (RAI) therapy is the mainstay treatment option for DTC patients with DMs. However, when patients with DMs are resistant to RAI, their 10-year survival rate is less than 10% (4). The ability of DMs to trap iodine is essential to the efficiency of RAI. The sodium iodide symporter (NIS) actively transports iodide from plasma against its concentration gradient (5). Therefore, the level of NIS expression and its correct location on cancer cells are very important for RAI accumulation.

NIS expression is regulated by genetic and epigenetic alterations. *BRAF*V600E is the most frequent genetic aberration in papillary thyroid cancer (PTC), occurring in 29%–83% of PTC but basically missing in follicular thyroid cancers (FTCs) (6). Transgenic mice with *BRAF*V600E mutation showed tumorigenic potential to rapidly and consistently develop PTC that in the majority of cases displayed a poorly differentiated phenotype with decreased NIS expression and radioiodine refractoriness (RAI-R) (7). Preclinical and clinical studies of *BRAF*V600E inhibitors (dabrafenib and vemurafenib) restored or enhanced partial lesions concentrating I-131 (8–10). However, *BRAF* mutation is genetically heterogeneous within PTC tumor cells, and the relationship between *BRAF*V600E mutation and the aggressiveness and prognosis of patients with PTC is controversial (11–13). We demonstrated that *BRAF*V600E mutation had no effect on radioiodine ablation and adjuvant therapy for PTC without DMs, similar to another study demonstrating that the clinical response to RAI therapy was not inferior in *BRAF*V600E mutation PTC patients without DM (11, 14). A recent clinical study demonstrated that the addition of selumetinib to adjuvant RAI failed to improve the complete response rate for PTC patients when compared with RAI alone (15).

When referring to DMs from PTC, *BRAF*V600E was demonstrated to influence the ability to accumulate RAI (16). However, the effect of *BRAF*V600E mutation on the prognosis of metastatic PTC patients needs to be further illustrated. Moreover, not all patients with *BRAF*V600E mutation of the primary tumor have lost the ability to accumulate RAI (16). Whether *BRAF*V600E mutation can affect clinical outcomes in metastatic PTC patients with radioiodine avidity and non-radioiodine avidity also needs to be clarified. Thus, this study was designed to assess the proportion of radioiodine avidity and the prognosis of lung metastatic PTC patients with *BRAF*V600E mutation of primary tumors and then compare them with DMs coming from non-*BRAF*V600E mutation PTC patients.

## Patients and methods

### Patients

During enrollment, 1,360 patients with *BRAF*V600E mutation testing results from our hospital from January 2012 to September 2021 were reviewed. The inclusion criteria were as follows: 1) patients with lung metastases (LMs) based on pathological biopsy-proven assessment, chest CT, or imaging of treatment dose whole-body scan; 2) no other distant metastases, such as bone, liver, and muscle; 3) patients older than 20 years at diagnosis. Ultimately, a total of 94 patients with LMs of histological confirmation as PTC with *BRAF*V600E mutation testing results of the primary tumor were retrospectively enrolled. All the patients were divided into a positive *BRAF*V600E mutation group and a negative *BRAF*V600E group according to the status of *BRAF*V600E mutation. In addition, all patients also were divided into the radioiodine avidity group and non-radioiodine avidity group according to the status of radioiodine uptake. The study was approved by our institutional review board, and the requirement for informed consent was waived.

The disease stages of PTC were defined according to the eighth edition of the American Joint Committee on Cancer TNM staging (17). Risk stratification was performed based on the 2015 American Thyroid Association guidelines (18).

### *BRAF*V600E mutational analysis

The method for determining the *BRAF*V600E mutation status of primary PTC tumors was described previously (14). Briefly, genomic DNA extracted from primary tumors was used to amplify the fragment of *BRAF* gene containing the T1799A hot spot. *BRAF*V600E was identified on sequencing electropherograms.

### Radioactive iodine therapy protocol

All patients underwent total thyroidectomy with at least central neck dissection and radioiodine therapy, followed by thyroid-stimulating hormone (TSH) suppression. One to six months after surgery, RAI therapy was administered according to the patients’ examination. Before each RAI therapy, to ensure that the TSH level was above 30 mIU/L, levothyroxine withdrawal was maintained for at least 3 weeks. For the dose of RAI, the fixed radioactivity of 3.7 to 7.4 GBq was administered, and 5.5 to 7.4 GBq was given to repeated RAI therapy for patients with radioiodine-avid LMs. Levothyroxine was given on the third day of radioiodine therapy. ^131^I whole-body scan was performed 3–5 days after radioiodine therapy. According to the results of the posttherapy ^131^I whole-body scan, PTC patients with radioiodine-avid LMs included patients in whom partial or all pulmonary nodules could accumulate radioiodine; non-radioiodine-avid LMs included those patients in whom no pulmonary nodules could accumulate radioiodine.

### Evaluation of therapeutic response

All patients were evaluated for LMs according to chest CT every 6 to 12 months. If there was at least one measurable pulmonary nodule (the longest diameter ≥1 cm), Response Evaluation Criteria in Solid Tumors (RECIST) 1.1 criteria (19) for target lesions were used to evaluate the therapeutic response as follows: 1) complete response (CR) if all lesions disappear and suppressed serum thyroglobulin level is undetectable, 2) partial response (PR) if the sum of the diameters of target lesions decreased more than 30%, 3) stable disease (SD) if the sum of the diameters of target lesions decreased less than 30% or increased less than 20%, and 4) progressive disease (PD) if new lesions developed or the sum of the diameters of target lesions increased more than 20% and absolute increase at least 5 mm. If there were no measurable pulmonary nodules, RECIST 1.1 criteria for non-target lesion was used, as follows: 1) CR if all lesions disappeared and the suppressed serum thyroglobulin level was undetectable, 2) non-CR/non-PD if one or more lesions existed persistently, and 3) PD if the number of lesions increased or there is an occurrence of distant metastases in other sites. Here, the PR and SD of measurable lesions were classified as non-CR/non-PD. Patients evaluated as CR or non-CR/non-PD were defined as non-PD.

Progression-free survival (PFS) was defined as the interval time between the first found LMs and the detection of PD. Overall survival (OS) was defined as the interval time from the time when LMs were first found to death.

### Statistical analysis

Statistical analysis was performed using SPSS 26.0 for Mac, Prism 9.0 was used for survival curves, a histogram was drawn in Excel, and an alluvial diagram was drawn by RStudio. The numeric variables are described as the mean, standard deviation, maximum, and minimum. Frequency or percentage is used to describe categorical variables. The chi-square test or Fisher’s test was used to identify the differences in subgroups in univariate analyses, and logistic regression was performed for multivariate analyses. PFS plots and OS plots were constructed by the Kaplan–Meier method. The alluvial diagram showed the relationship between *BRAF*V600E mutation status, radioiodine avidity, and clinical outcome. p < 0.05 was considered statistically significant.

## Results

### All patient characteristics

Ninety-four PTC patients identified with LMs from January 2012 to September 2021 met the inclusion criteria. The median age of all patients was 42 years (ranging from 20 to 77 years), and 70.2% (66/94) of them were female (**Table 1**).

**Table 1:** Clinical characteristics of all patients.

Characteristics	N
Age (years)	
≥55	22 (23.4%)
<55	72 (76.6%)
Gender	
Male	28 (29.8%)
Female	66 (70.2%)
Multifocality	
Yes	26 (27.7%)
No	55 (58.5%)
NA	13 (13.8%)
Bilaterality	
Yes	34 (36.2%)
No	52 (55.3%)
NA	8 (8.5%)
CI	
Yes	72 (76.6%)
No	5 (5.3%)
NA	17 (18.1%)
ETE	
Yes	54 (57.5%)
No	27 (28.7%)
NA	13 (13.8%)
T stage*	
T1/T2/T3	40 (42.6%)
T4	35 (37.2%)
Tx**^†^**	19 (20.2%)
N stage*	
N0	0 (0.0%)
N1a	12 (12.8%)
N1b	77 (81.9%)
Nx**^†^**	5 (5.3%)
*BRAF*V600E	
Positive	33 (35.1%)
Negative	61 (64.9%)
Radioiodine uptake	
Radioiodine-avid	45 (47.9%)
Non-radioiodine-avid	49 (52.1%)

CI, capsule invasion; NA, non-available; ETE, extrathyroidal extension.

^*^TNM staging was determined by the 8th American Joint Cancer Committee TNM stage system.

^†^Indicates that information about that characteristic was not available.

**Figure 1:**
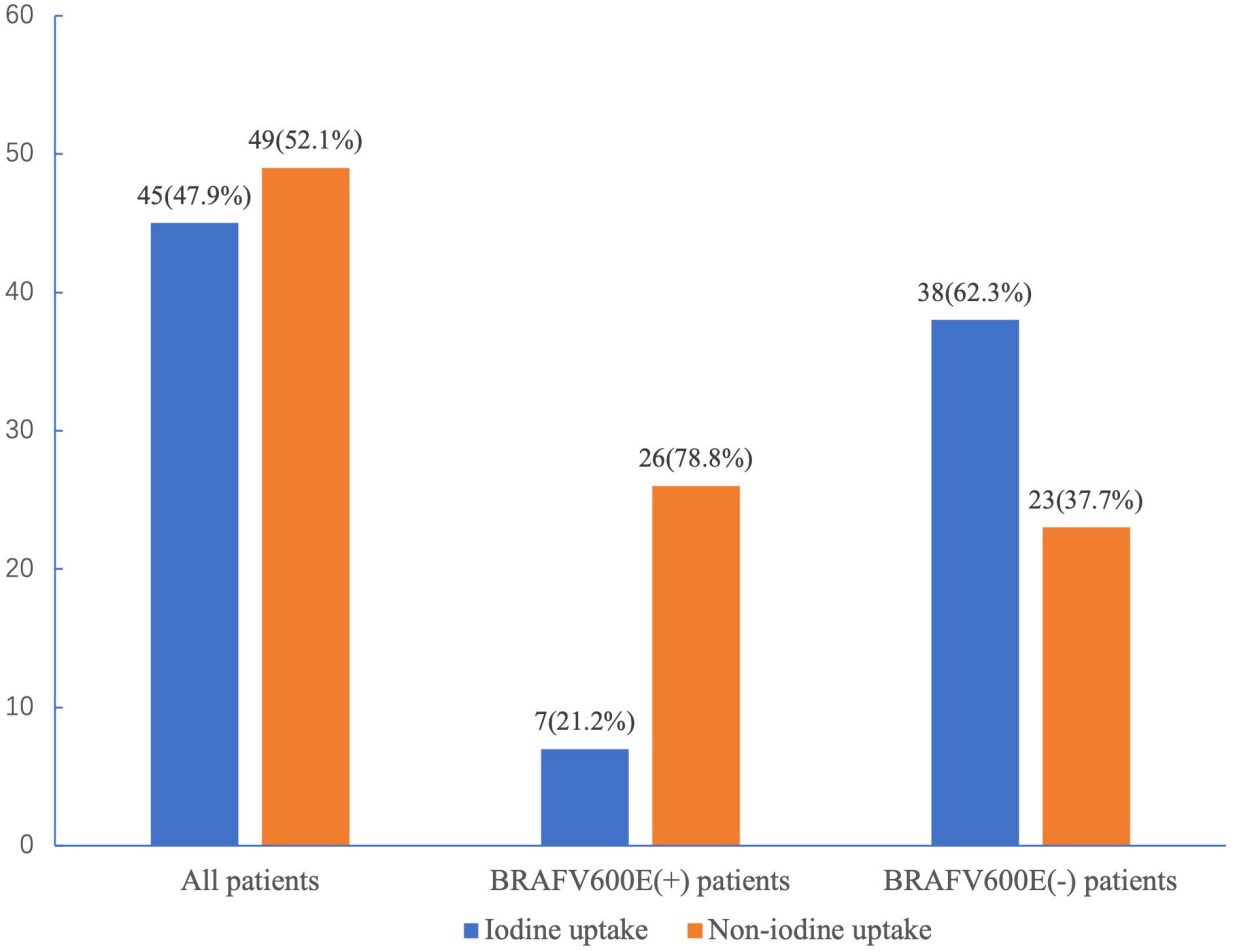
*BRAF*V600E mutation status and radioiodine avidity status in all patients.

According to the *BRAF*V600E mutation testing results, 33 patients with a median age of 51 years (25–77 years) were assigned to the positive *BRAF*V600E mutation group, and 61 patients with a median age of 37 years (20–69 years) were assigned to the negative *BRAF*V600E group. The overall prevalence of *BRAF*V600E mutation was 35.1% (33/94). As shown in **Figure 1**, in the positive *BRAF*V600E mutation group, 21.2% (7/33) of patients had radioiodine-avid LMs, and 78.8% (26/33) of patients had non-radioiodine-avid LMs, while in the negative *BRAF*V600E group, 62.3% (38/61) of patients had radioiodine-avid LMs, and 37.7% (23/61) of patients had non-radioiodine-avid LMs.

### Factors impacting the status of radioiodine avidity

According to the status of radioiodine avidity, 49 and 45 patients were assigned to the non-radioiodine avidity group and radioiodine avidity group, respectively. The characteristics, including age, sex, multifocality, capsular invasion, bilaterality, extrathyroidal extension (ETE), TN stage, and the status of *BRAF*V600E mutation, were evaluated for their effects on radioiodine avidity. All characteristics were independent of radioiodine avidity except age and the status of *BRAF*V600E mutation, as shown in **Table 2**. Meanwhile, positive *BRAF*V600E mutation was the only predictor of radioiodine avidity loss in multivariate analysis; the odds ratios (ORs) were 5.323 (95% CI: 1.953–14.514, p = 0.001, **Table 2**).

**Table 2:** The relationship between clinical features and the loss of radioiodine avidity.

Characteristics	Non-radioiodine-avid	Radioiodine-avid	χ^2^	p	OR	p
No. patients	49 (52.1%)	45 (47.9%)				
Age (years)			4.884	0.027		
≥55	16 (72.7%)	6 (27.3%)			1	
<55	33 (45.8%)	39 (54.2%)			2.146 (0.691–6.661)	0.186
Gender			0.402	0.526		
Male	16 (57.1%)	12 (42.9%)				
Female	33 (50.0%)	33 (50.0%)				
Multifocality			2.366	0.124		
Yes + NA	24 (61.5%)	15 (38.5%)				
No	25 (45.5%)	30 (54.5%)				
CI			0.000	1.000		
Yes + NA	46 (51.7%)	43 (48.3%)				
No	3 (60.0%)	2 (40.0%)				
Bilaterality			2.908	0.088		
Yes + NA	26 (61.9%)	16 (38.1%)				
No	23 (44.2%)	29 (55.8%)				
ETE			0.240	0.624		
Yes + NA	36 (53.7%)	31 (46.3%)				
No	13 (48.1%)	14 (51.9%)				
T stage			0.011	0.917		
T1/T2/T3/Tx	31 (52.5%)	28 (47.5%)				
T4	18 (51.4%)	17 (48.6%)				
N stage			0.214	0.644		
N1a/Nx	8 (47.1%)	9 (52.9%)				
N1b	41 (53.2%)	36 (46.8%)				
*BRAF*V600E			14.484	0.000		
Positive	26 (78.8%)	7 (21.2%)			1	
Negative	23 (37.7%)	38 (62.3%)			5.323 (1.953–14.514)	0.001

CI, capsule invasion; NA, non-available; ETE, extrathyroidal extension.

### Clinical characteristics between positive and negative *BRAF*V600E patients in radioiodine-avid and non-radioiodine-avid subgroups

In the radioiodine avidity subgroup of 45 patients, there were no significant differences in clinicopathological characteristics between the positive and negative *BRAF*V600E mutation groups (**Table 3**). In the non-radioiodine avidity subgroup, the median age of patients with positive *BRAF*V600E mutation was older than that in the mutation-negative group (53.5 *vs.* 35.0 years, Z = −3.629, p = 0.000); then, no significant differences in other clinicopathological characteristics between patients with positive and negative *BRAF*V600E mutation were found (**Table 3**).

**Table 3:** Clinical characteristics of *BRAF*V600E(+) and *BRAF*V600E(−) patients in radioiodine avidity subgroup and non-radioiodine avidity subgroup.

Characteristics	Radioiodine avidity			Non-radioiodine-avidity		
	*BRAF*V600E (+)	*BRAF*V600E (−)	p	*BRAF*V600E (+)	*BRAF*V600E (−)	p
No. patients	7 (15.6%)	38 (84.4%)		26 (53.1%)	23 (46.9%)	
Age (years)			1.000			0.032
≥55	1 (16.7%)	5 (83.3%)		12 (75.0%)	4 (25.0%)	
<55	6 (15.4%)	33 (84.6%)		14 (42.4%)	19 (57.6%)	
Gender			0.129			0.765
Male	4 (33.3%)	8 (66.7%)		8 (50.0%)	8 (50.0%)	
Female	3 (9.1%)	30 (90.9%)		18 (54.5%)	15 (45.5%)	
Multifocality			1.000			0.321
Yes/NA	2 (13.3%)	13 (86.7%)		11 (45.8%)	13 (54.2%)	
No	5 (16.7%)	25 (83.3%)		15 (60.0%)	10 (40.0%)	
CI			1.000			1.000
Yes/NA	7 (16.3%)	36 (83.7%)		24 (52.2%)	22 (47.8%)	
No	0 (0.0%)	2 (100.0%)		2 (66.7%)	1 (33.3%)	
Bilaterality			1.000			0.303
Yes/NA	2 (12.5%)	14 (87.5%)		12 (46.2%)	14 (53.8%)	
No	5 (17.2%)	24 (82.8%)		14 (60.9%)	9 (39.1%)	
ETE			1.000			0.947
Yes/NA	5 (16.1%)	26 (83.9%)		19 (52.8%)	17 (47.2%)	
No	2 (14.3%)	12 (85.7%)		7 (53.8%)	6 (46.2%)	
T stage			1.000			0.390
T1/T2/T3/Tx	4 (14.3%)	24 (85.7%)		15 (48.4%)	16 (51.6%)	
T4	3 (17.6%)	14 (82.4%)		11 (61.1%)	7 (38.9%)	
N stage			0.918			0.564
N1a/Nx	2 (22.2%)	7 (77.8%)		3 (37.5%)	5 (62.5%)	
N1b	5 (13.9%)	31 (86.1%)		23 (56.1%)	18 (43.9%)	
Cycles of RAI	2 (1–3)	1 (1–5)	0.304	NA	NA	NA
Cumulate dose of ^131^I (GBq)	14.8 (3.7–22.2)	7.4 (3.7–37.0)	0.487	NA	NA	NA

CI, capsule invasion; NA, non-available; ETE, extrathyroidal extension; RAI, radioactive iodine.

### Predictors of progressive disease of papillary thyroid cancer patients with lung metastases in univariate and multivariate analyses

**Figure 2:**
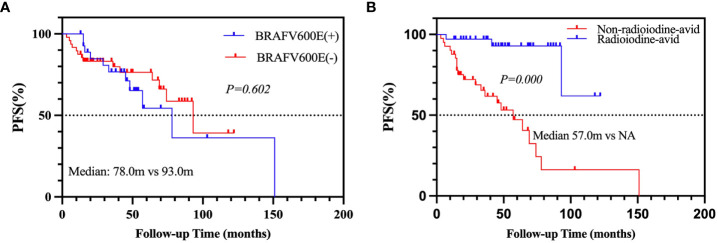
Kaplan–Meier analysis of PFS in PTC patients according to *BRAF*V600E mutation status **(A)** and radioiodine avidity status **(B)**. PFS, progression-free survival; PTC, papillary thyroid cancer.

After a median 29-month (range from 3 to 151 months) follow-up, 25 patients (26.6%) were defined as PD, and 51 patients (54.3%) were defined as non-PD. Eighteen (19.1%) patients were not evaluated due to a follow-up time of less than 12 months or an inability to assess LMs. Consequently, 76 PTC patients were enrolled in the analysis; the cumulative PFS rates at 5 and 10 years were 68.7% and 41.0%, respectively. Univariate analyses found that the status of *BRAF*V600E mutation was not significantly associated with PD (**Table 4**, p = 0.365), although the median PFS of positive *BRAF*V600E was shorter than that of negative *BRAF*V600E (78.0 *vs.* 93.0 m; **Figure 2A**, p = 0.602), whereas the status of radioiodine avidity and bilaterality of primary tumor were the predictors of PD. In the multivariate analyses, the status of radioiodine avidity was only the independent predictor of PD (ORs: 10.207, 95% CI: 2.629–39.643, p = 0.001), as shown in **Table 4**. The median PFS of the non-radioiodine avidity group *vs.* radioiodine avidity group was 57.0 m *vs.* not reached (log-rank = 18.256, p = 0.000), as shown in **Figure 2B**.

**Table 4:** Univariate and multivariate analyses of predictors of progressive disease in 76 patients.

Characteristics	No. of PD	χ^2^	p	OR	p
No. patients	25 (32.9%)				
Age (years)		3.126	0.077		
≥55	9 (50.0%)			1	
<55	16 (27.6%)			0.554 (0.159–1.935)	0.355
Gender		0.901	0.343		
male	9 (40.9%)				
female	16 (29.6%)				
Multifocality		1.529	0.216		
Yes + NA	12 (41.4%)				
No	13 (27.7%)				
CI		–	1.000		
Yes + NA	24 (32.9%)				
No	1 (33.3%)				
Bilaterality		4.168	0.041		
Yes + NA	15 (45.5%)			1	
No	10 (23.3%)			0.502 (0.164–1.532)	0.226
ETE		0.016	0.899		
Yes + NA	18 (33.3%)				
No	7 (31.8%)				
T stage		1.168	0.280		
T1/T2/T3/Tx	14 (28.6%)				
T4	11 (40.7%)				
N stage		0.004	0.947		
N1a/Nx	4 (28.6%)				
N1b	21 (33.9%)				
*BRAF*V600E		0.820	0.365		
Positive	11 (39.3%)				
Negative	14 (29.2%)				
Radioiodine uptake		17.388	0.000		
Radioiodine-avid	3 (8.6%)			1	
Non-radioiodine-avid	22 (53.7%)			10.207 (2.629–39.643)	0.001

PD, progressive disease; CI, capsule invasion; NA, non-available; ETE, extrathyroidal extension.

In addition, a subgroup analysis in the radioiodine avidity group and non-radioiodine avidity group was used to analyze the relationship between *BRAF*V600E mutation status and PD. In the radioiodine avidity subgroup, all positive *BRAF*V600E patients (7/7) remained non-CR/non-PD; as for patients with *BRAF*V600E negative mutation, the rates of CR, non-CR/non-PD, and PD were 0% (0/28), 89.3% (25/28), and 10.7% (3/28), respectively (p = 1.000, **Table 5**). Similarly, in the non-radioiodine avidity subgroup, no patients achieved CR. The rate of non-CR/non-PD and PD in positive and negative *BRAF*V600E mutation patients were 47.6% (10/21) and 52.4% (11/21) *vs.* 45.0% (9/20) and 55.0% (11/20), respectively (p = 0.867, **Table 5**).

**Table 5:** Association between progressive disease and *BRAF*V600E mutation status in radioiodine avidity subgroup and non-radioiodine avidity subgroup.

*BRAF*V600E mutation	Clinical outcome							
	Radioiodine avidity				Non-radioiodine-avidity			
	CR	Non-CR/non-PD	PD	p	CR	Non-CR/non-PD	PD	p
Positive	0 (0.0%)	7 (100.0%)	0 (0.0%)	1.000	0 (0.0%)	10 (47.6%)	11 (52.4%)	0.867
Negative	0 (0.0%)	25 (89.3%)	3 (10.7%)		0 (0.0%)	9 (45.0%)	11 (55.0%)	

CR, complete response; PD, progressive disease.

**Figure 3:**
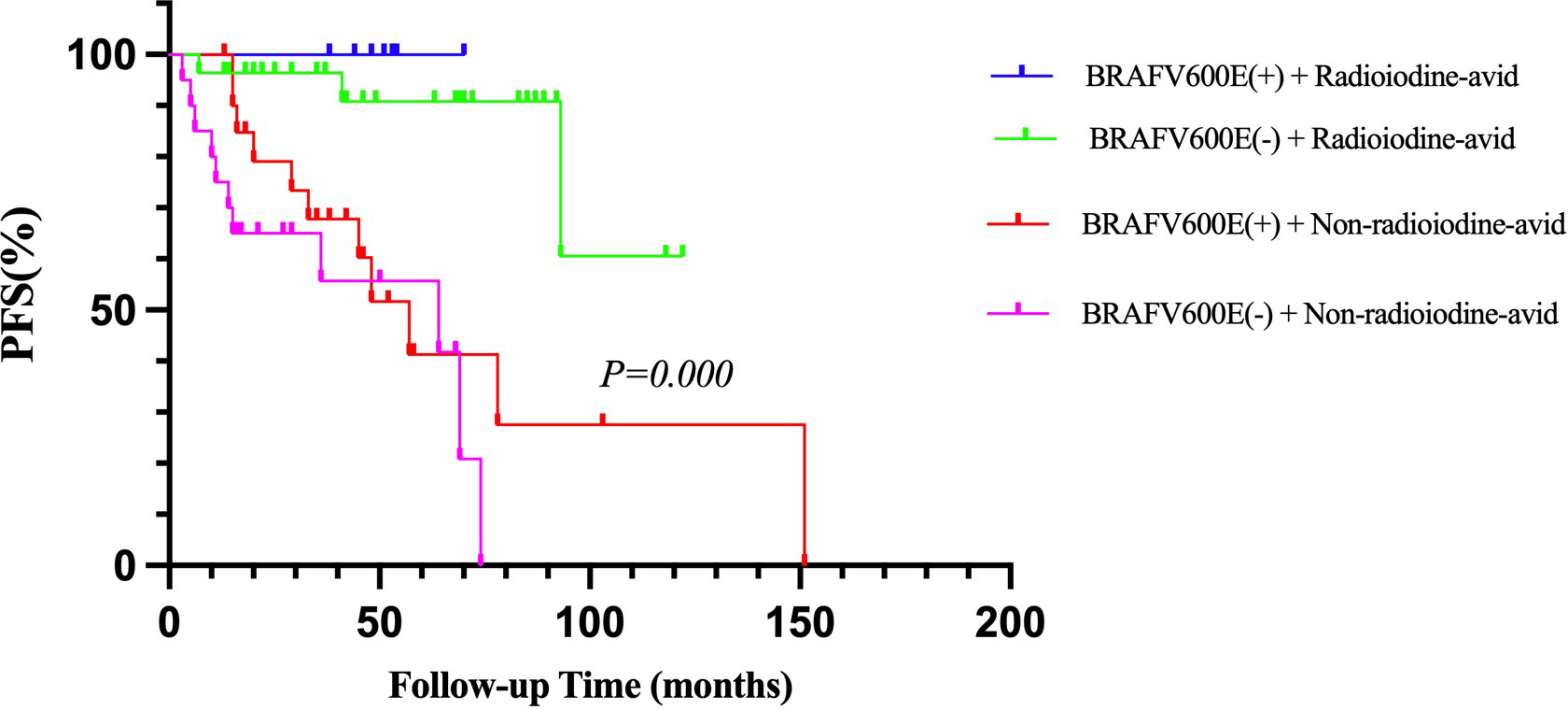
Kaplan–Meier analysis of PFS in PTC patients according to *BRAF*V600E mutation status combined with radioiodine avidity status. PFS, progression-free survival; PTC, papillary thyroid cancer.

**Figure 4:**
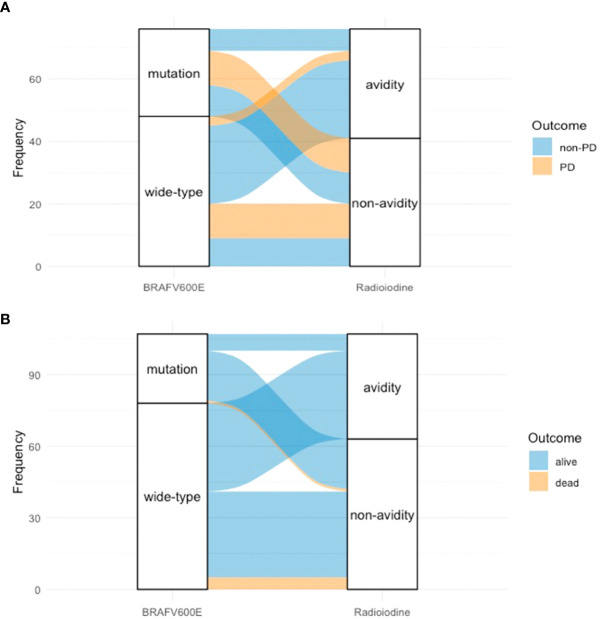
Alluvial diagram of PTC patients showed the relationship between *BRAF*V600E mutation status, radioiodine avidity status, and outcome. **(A)** *BRAF*V600E mutation status, radioiodine avidity status, and PD. **(B)** *BRAF*V600E mutation status, radioiodine avidity status, and mortality. PTC, papillary thyroid cancer; PD, progressive disease.

When combining the status of *BRAF*V600E mutation and the status of radioiodine avidity into the survival analysis of PFS, we found that patients with positive *BRAF*V600E mutation and non-radioiodine-avid LMs had the shortest median PFS (p = 0.000, **Figure 3**). The relationship among *BRAF*V600E mutation status, radioiodine avidity, and PD were shown by an alluvial diagram (**Figure 4A**).

### Predictors of mortality of papillary thyroid cancer patients with lung metastases in univariate analyses

**Figure 5:**
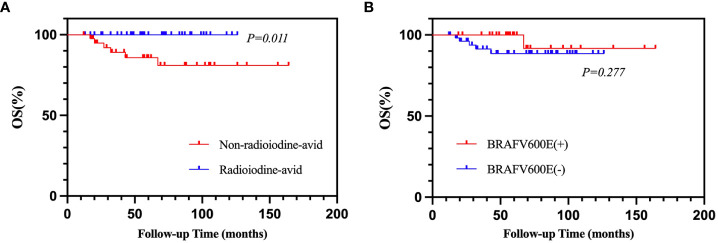
Kaplan–Meier analysis of OS in PTC patients according to radioiodine avidity status **(A)** and *BRAF*V600E mutation status **(B)**. OS, overall survival; PTC, papillary thyroid cancer.

Of 94 patients, eight were excluded from the analysis due to a follow-up time of less than 12 months. Therefore, 86 patients were enrolled in the analysis. After a median 56.5-month (range from 12 to 164 months) follow-up time, the mortality rate of all patients was 7.0% (6/86). The cumulative 5- and 10-year OS rates were 92.9% and 90.4%, respectively. Applying the chi-square test for the univariate analyses of death rate (**Table 6**), the mortality rates of non-radioiodine-avid patients and radioiodine-avid patients were 14.3% (6/42) and 0.0% (0/44), respectively (χ^2^ = 4.735, p = 0.030). In total, 1/29 (3.4%) and 5/57 (8.8%) mortalities were observed in the positive BRAFV600E mutation patients and mutation-negative patients, respectively (χ^2^ = 0.219, p = 0.639). The status of iodine uptake was the only factor associated with the death rate. Similarly, patients with radioiodine-avid LMs may have longer OS than that non-radioiodine avidity LMs, although they did not achieve the median OS (p = 0.011, **Figure 5A**). The status of *BRAF*V600E mutation did not influence the OS of PTC patients (p = 0.277, **Figure 5B**). An alluvial diagram that showed the correlation among *BRAF*V600E mutation status, radioiodine avidity, and PD is listed in **Figure 4B**.

**Table 6:** Univariate chi-square analysis of prognostic factors of mortality in 86 patients.

Characteristics	No. of death	χ^2^	p
No. patients	6 (7.0%)		
Age (years)		0.001	0.973
≥55	2 (9.5%)		
<55	4 (6.2%)		
Gender		2.679	0.102
Male	4 (16.0%)		
Female	2 (3.3%)		
Multifocality		0.012	0.912
Yes + NA	3 (8.8%)		
No	3 (5.8%)		
CI		–	1.000
Yes + unknown	6 (7.4%)		
No	0 (0.0%)		
Bilaterality		0.719	0.396
Yes + NA	4 (11.1%)		
No	2 (4.0%)		
ETE		1.467	0.226
Yes + NA	6 (10.0%)		
No	0 (0.0%)		
T stage		1.390	0.238
T1/T2/T3/Tx	2 (3.6%)		
T4	4 (12.9%)		
N stage		0.174	0.676
N1a/Nx	2 (12.5%)		
N1b	4 (5.7%)		
*BRAF*V600E		0.219	0.639
Positive	1 (3.4%)		
Negative	5 (8.8%)		
Radioiodine uptake		4.735	0.030
Radioiodine-avid	0 (0.0%)		
Non-radioiodine-avid	6 (14.3%)		

CI, capsule invasion; NA, non-available.

## Discussion

*BRAF*V600E mutation is the most common genetic alteration in thyroid carcinogenesis, which occurs in approximately 45% of PTC patients (6). However, a previous study by Sancisi et al. (20) found that 29.8% of PTC patients with DMs and 44.0% of PTC patients without DMs had positive *BRAF*V600E mutations. In another study by Yang et al. (16), there were 26.0% of metastatic PTC patients with positive *BRAF*V600E mutation. Similarly, 35.1% of metastatic PTC patients had positive *BRAF*V600E mutation in our study, and 66.0% of patients without DMs had positive *BRAF*V600E mutation in our previous study (14). These results indicated that PTC patients with DMs may have a lower mutation frequency of *BRAF*V600E than patients without DMs.

*BRAF*V600E mutation has been found to repress NIS expression by impairing thyroid-specific transcription factor (PAX8) binding to the NIS promotor by activating transforming growth factor-β/Smad3 signaling. It was also demonstrated that this mutation prevents NIS transcription by driving histone deacetylation of the H3 and H4 lysine residues of the NIS (21–23). The Cancer Genome Atlas (TCGA) sequencing divided PTC into *BRAF*-like and RAS-like groups. *BRAF*-like PTC is associated with reduced expression of key genes involved in iodine metabolism (24). Based on these molecular mechanisms, many previous studies have explored the relationship between *BRAF*V600E mutation and the ability to trap iodine in PTC. A study by Durante et al. (25) found that the transcript level of positive *BRAF*V600E mutation patients reduced NIS expression by 82% compared to wild-type *BRAF*V600E patients. They assumed that the *BRAF*V600E mutation in PTC altered the effectiveness of radioiodine therapy. Similarly, Ricarte et al. (26) also revealed that RAI-refractory metastatic thyroid cancers are enriched with *BRAF*V600E mutation (62%). A respective study by Liu et al. (27) revealed higher RAI avidity loss in the positive *BRAF*V600E mutation group than in the wild-type group (80.4% *vs.* 33.9%). In addition, Yang et al. (16) reported that 93.4% of distant metastases in PTC patients with positive *BRAF*V600E mutation of primary tumors were more likely to be non-radioiodine avidity. In our study, positive *BRAF*V600E mutation patients are also more likely to lose radioiodine avidity than negative *BRAF*V600E mutation patients (78.8% *vs.* 37.7%); ORs were 5.323 (95% CI: 1.953–14.514).

On the one hand, we found that not all patients with positive *BRAF*V600E mutation lost radioiodine avidity, as 21.2% of patients retained RAI uptake in our study. Further evaluation of the clinical outcome in *BRAF*V600E mutation-negative and *BRAF*V600E mutation-positive patients respectively in the radioiodine-avid and non-radioiodine-avid LM subgroups found no significant differences between those groups. TCGA sequencing also showed that partial positive *BRAF*V600E mutation patients reserved the expression of NIS transcription (28). This may be due to tumor heterogeneity, in which primary PTC tumors were composed of a mixture of positive *BRAF*V600E mutation tumor cells and negative *BRAF*V600E mutation tumor cells, and the metastatic tumor did not genetically gain *BRAF*V600E mutation (29). Quantitative sequencing analyses demonstrated that *BRAF*V600E mutation was detected only in a subset of tumor cells (approximately 5.1% to 44% of total alleles) (30). Another reason is that in all of the above studies, the gene results of primary tumors were used for analysis; however, in the Melo et al. (31) study, when comparing the gene concordance of primary tumors and DMs, they found significant discrepancies in primary PTC and DMs, with mutation frequencies of 44.6% and 23.8%, respectively.

On the other hand, in this study, 37.7% of patients with negative *BRAF*V600E mutation could not accumulate radioiodine. We assumed the reason may be that other gene aberrations exist, such as RAS mutation and RET rearrangements of thyroid cancer, which have been reported to activate the MAPK pathway, followed by the dedifferentiation of DTCs (32). Liu et al. (27) demonstrated that 55.6% of patients with TERT mutation alone would be non-radioiodine-avid. In addition, metastatic lesions have a median of 62% somatic mutations corresponding to primary tumor samples that do not have (33).

The effect of *BRAF*V600E mutation on the prognosis of PTC patients is controversial. Many studies have illustrated the relationship between *BRAF*V600E mutation and the prognosis of PTC patients. A 15-year median follow-up study by Elisei et al. (34) that included 102 PTC patients found that *BRAF*V600E mutation was an independent factor correlated with the worst outcomes in a higher risk of not being cured and death. In a multicenter study of 219 PTC patients, Xing et al. (35) found that positive *BRAF*V600E mutation may be associated with the recurrence of tumors (25% *vs.* 9%); this study also indicated that PTC patients with positive *BRAF*V600E mutation may have a worse prognosis than those patients with a negative mutation. Another retrospective and multicenter study including 1,849 patients by Xing et al. (13) revealed that the presence of *BRAF*V600E mutation was associated with an increased mortality rate in PTC patients based on the difference in mortality rate in *BRAF*V600E-positive mutation patients and *BRAF*V600E-negative mutation patients (5.3% *vs.* 1.1%). However, when clinicopathological features such as lymph node metastases, extrathyroidal extension, and distant metastases were included in the analysis model, *BRAF*V600E mutation was no longer associated with mortality caused by PTC. A meta-analysis by Vuong et al. (36) including 35 studies with 17,732 patients revealed that *BRAF*V600E mutation was significant in a short-term follow-up because *BRAF*V600E mutation increased the risk of recurrence (HR = 1.63; 95% CI = 2.40–3.96) but was independent of cancer mortality rate (HR = 1.41; 95% CI = 0.90–2.23).

In our study, we found that *BRAF*V600E mutation-positive patients had shorter PFS than *BRAF*V600E mutation-negative patients, but there were no significant differences (median: 78.0 *vs.* 93.0 m in the positive group *vs.* the negative group). At the same time, we found that positive *BRAF*V600E mutation was also not associated with OS in metastatic PTC patients, although neither reached a median OS due to the short follow-up time. The mortality rate of all patients was 7.0% in our study, which was consistent with the reported result (13). The findings of Pu et al. (24) may explain the controversy regarding *BRAF*V600E mutation in predicting the prognosis of PTC by using single-cell transcriptomic analysis and refining bulk molecular subtyping. *BRAF*-like patients can be further divided into *BRAF*-like-A and *BRAF*-like-B subclasses, and *BRAF*-like-B patients had a lower thyroid differentiation score, advanced staging, and a significantly compromised prognosis (24). In our study, we found that the status of radioiodine avidity was associated with clinical outcome and *BRAF*V600E mutation was one of the factors influencing radioiodine avidity. However, not all patients with positive *BRAF*V600E mutation lost the ability to uptake iodine, and not all patients with negative *BRAF*V600E mutation reserved the ability to accumulate iodine. Therefore, a relationship between *BRAF*V600E mutation and prognosis was not found in our study.

The study has several limitations. First, the small number of PTC patients with LMs and *BRAF*V600E mutation results may influence the accuracy of this study. Second, *BRAF*V600E mutation testing results of primary PTC rather than LMs were used for evaluations due to limited sources. Third, selection bias may be caused by the retrospective nature of the study. Fourth, we only enrolled PTC patients with LMs and excluded patients with metastases in other sites that may not represent DMs. Fifth, the follow-up time is short for PTC patients. In addition, multiple pulmonary nodules were so small that the lesion measurements may be inaccurate.

## Conclusion

In our study, we found that although the *BRAF*V600E mutation status of primary tumors was significantly associated with non-radioiodine-avid LMs in patients with PTC, *BRAF*V600E mutation status may not influence the prognosis of PTC patients with LMs. Radioiodine avidity of LMs was the only independent prognostic factor of clinical outcome. A longer follow-up needs to be undertaken to identify the predictors associated with OS of PTC patients with LMs.

## Data availability statement

The original contributions presented in the study are included in the article/supplementary material. Further inquiries can be directed to the corresponding authors.

## Ethics statement

The studies involving human participants were reviewed and approved by Ethics Institutional Review Board of West China hospital of Sichuan University. Written informed consent for participation was not required for this study in accordance with the national legislation and the institutional requirements.

## Author contributions

SH and MQ collected clinical data and drafted the article. They are equally contributed to this article. TT and HD analyzed the data. RH and YT performed conception of the work, critical revision of the article and final approval of the version to be published. All authors contributed to the article and approved the submitted version.

## Funding

This study was supported by the National Natural Science Foundation of China (No. 81972502).

## Conflict of interest

The authors declare that the research was conducted in the absence of any commercial or financial relationships that could be construed as a potential conflict of interest.

## Publisher’s note

All claims expressed in this article are solely those of the authors and do not necessarily represent those of their affiliated organizations, or those of the publisher, the editors and the reviewers. Any product that may be evaluated in this article, or claim that may be made by its manufacturer, is not guaranteed or endorsed by the publisher.
